# Macrophage heterogeneity in tissues: phenotypic diversity and functions

**DOI:** 10.1111/imr.12223

**Published:** 2014-10-15

**Authors:** Siamon Gordon, Annette Plüddemann, Fernando Martinez Estrada

**Affiliations:** 1 Sir William Dunn School of Pathology, University of OxfordOxford, UK; 2 Nuffield Department of Primary Care Health Sciences, University of OxfordOxford, UK; 3 Botnar Research Centre, NDORMS, University of OxfordOxford, UK

**Keywords:** Tissue macrophages, monocytes, macrophages, heterogeneity, phenotype, markers

## Abstract

During development and throughout adult life, macrophages derived from hematopoietic progenitors are seeded throughout the body, initially in the absence of inflammatory and infectious stimuli as tissue-resident cells, with enhanced recruitment, activation, and local proliferation following injury and pathologic insults. We have learned a great deal about macrophage properties *ex vivo* and in cell culture, but their phenotypic heterogeneity within different tissue microenvironments remains poorly characterized, although it contributes significantly to maintaining local and systemic homeostasis, pathogenesis, and possible treatment. In this review, we summarize the nature, functions, and interactions of tissue macrophage populations within their microenvironment and suggest questions for further investigation.

This article is part of a series of reviews covering Monocytes and Macrophages appearing in Volume 262 of *Immunological Reviews*.

## Introduction

Macrophages are remarkably versatile in their ability to recognize and respond to a wide range of stimuli, expressing a variety of surface and intracellular receptors, multiple signal transduction pathways and complex, adaptable arrays of gene expression. They are long-lived, biosynthetically active cells with potent endocytic, phagocytic, and secretory functions, able to modulate their properties upon contact with different cell types as well as extracellular matrix. Their intrinsic heterogeneity during differentiation is compounded by reciprocal interactions with neighboring cells, including macrophages themselves, diverse microorganisms, sterile particulates and soluble mediators. We summarize general features of the mononuclear phagocyte system, before dealing with diversity of macrophages in specific locations. We emphasize early studies with membrane antigen markers, mainly in the mouse, to provide a foundation for contemporary analysis of global gene expression analysis, in both human and mouse.

## General considerations

Most of our knowledge regarding tissue macrophages is derived from mouse studies 1,2. Mouse tissue macrophages originate from precursors in hematopoietic organs, e.g. yolk sac and fetal liver in the embryo, and bone marrow and other hematopoietic sites such as spleen, postnatally 3–6. The nature of the cellular receptors and chemotactic signals that guide their constitutive migration within tissues remains obscure. Within tissues, macrophages have been traditionally described as sessile, or ‘fixed’; however, their further migration can be enhanced by inflammatory stimuli, especially chemokines generated locally by humoral and cellular mechanisms.

In response to inflammation and infection, bone marrow-derived blood-borne monocytes give rise to cells that remain within the circulation or enter tissues, giving rise to both macrophages and dendritic cells (DCs) 7; ‘elicited’ macrophages often show enhanced turnover at local sites 8. Recruited monocytes and sinus-lining tissue macrophages interact with blood, lymph, and extracellular fluid, as well as every cell type, including lympho-hematopoietic cells, endothelium, epithelium, mesenchymal, and neuronal cells. They express receptors and ligands for the products of resting and activated immune and non-immune cells, interacting through cell–cell contact, secretion, and endocytosis. The resident and newly recruited populations of macrophages at particular locations result from a dynamic process of production and entry, local proliferation, senescence, death, and emigration; recent studies have rediscovered the importance of local proliferation and self-renewal, dependent on growth factors such as M-CSF, GM-CSF and cytokines including IL-4/IL-13 and IL-6 9,10. The macrophage phenotypic changes that accompany residence in different tissues are still poorly understood.

The effects of heterogeneous macrophage populations on their local tissue environment include trophic as well as cytocidal interactions with neighboring cells, the remodeling of matrix, as well as phagocytic clearance of dying cells and other homeostatic and defense functions. These involve non-phagocytic intercellular adhesion, release and capture of secretory products, microvesicles and exosomes, nanotube formation, and cell fusion. Macrophage-derived secretory molecules include enzymes such as lysozyme, neutral proteinases, pro-and anti-inflammatory cytokines, chemokines and growth factors, arachidonate, oxygen, and nitrogen-derived metabolites.

Macrophages are metabolically active, influencing the functions of local and systemic target tissues; they are responsive to purinergic ligands, hormones, and vitamins, and are sensitive to oxygen and ionic changes in their immediate environment. Their role in heme and iron metabolism and recycling are considered below. Tissue macrophages are embedded in extracellular matrix, express receptors for collagens, elastin, proteoglycans, and reticular/fibrillar constituents, including bound cytokines, and in turn generate potent extracellular proteinases and anti-proteinase inhibitors to modulate matrix, cellular, and plasma protein functions.

Macrophages express a range of opsonic and non-opsonic membrane receptors for uptake and detection of microbial, sterile, and altered self-ligands; these include FcR, Complement R, TLR, lectins, and Scavenger receptors 11. Cytoplasmic sensors include Nod-like and RIG-I-like helicases and cyclic dinucleotides. The effects of macrophage activation on plasma membrane, endocytic and cytosolic recognition and sensing mechanisms, signal transduction, and altered gene expression have been extensively documented 12,13.

Aside from differentiation, determined by lineage-determining transcription factors such as PU-1, chromatin organization and epigenetic mechanisms, extrinsic stimuli such as infection elicit dynamic changes in gene transcription, loosely known as cell activation 14,15. Based on a few signals, these have been classified as ‘innate’ (TLR),’classical' (Th1/M1-type), and ‘alternative’ (Th2/M2-type) activation 14, but are now thought to constitute a more complex multipolar spectrum of gene expression signatures 16,17. A more realistic paradigm, extending it to the complexity of ligands present in different tissues and inflammatory conditions, is urgently needed.

For the present, the questions arise, how is the phenotype of macrophages influenced by different tissue environments, and what are the effects of macrophage activation on the particular tissue in which they reside? A further issue is whether organ-specific differences persist after macrophage activation by inflammation, infection, and malignancy. We consider these questions with particular reference to selected resident and recruited macrophage populations and in their response to granuloma formation.

## Evidence for tissue macrophage heterogeneity

Since the early studies by light and intravital microscopy of invertebrates as well as mammals by developmental biologists/experimental pathologists such as Metchnikoff, it was apparent that macrophage-like cells were dispersed in many tissues, performing diverse homeostatic and defense functions 18. The concept of a reticulo-endothelial system of phagocytic cells (RES) associated with Aschoff, emphasized the clearance functions of sinus-lining cells in liver, spleen, as well as in selected endocrine organs. Regional macrophages and related cells were also described in liver (Kupffer cells), lung (alveolar macrophages), and brain (microglia). Transmission and scanning electron microscopy of lympho-hematopoietic tissues emphasized the morphologic heterogeneity of monocytes and macrophages in different organs. From work on the spleen, it became apparent that macrophages could occupy different anatomical niches and perform specialized functions even within the same organ.

In 1968, a group of investigators proposed the term ‘mononuclear phagocyte system’ to unify diverse populations of macrophage-like cells; however, there was still considerable confusion about which properties could be used to define the cells belonging to this extended family, as well as their origins, relationships, activation, and functional diversity 19. Subsequent development of monoclonal antibodies directed against membrane antigens made an enormous impact on the field, followed by cellular biology and molecular genetic techniques to manipulate and establish precursor-product and lineage relationships.

The group of Alan Williams and Neil Barclay, at Oxford 20, utilized a systematic approach to produce and characterize a panel of anti-lymphocyte monoclonal antibodies of which several also reacted with myeloid cells. These have been particularly useful in characterizing rat lymphoid subsets, their localization and functions. In our own laboratory, we initially sought macrophage-specific reagents for mouse macrophages. The rat anti-mouse reagent F4/80, subsequently shown to recognize an adhesion GPCR EMR1, was particularly useful for immunocytochemical definition of macrophages during development, in the normal and challenged adult mouse, and drew our attention to tissue macrophage diversity in antigen expression 21,22. The F4/80 epitope was selected to be stable to perfusion fixation and is mainly expressed on the plasma membrane, thus proving ideal to establish macrophage interactions with neighboring cells 23,24. From these studies it became possible to delineate F4/80+ populations, which were associated with epithelia, endothelia and connective tissue, as well as free cells in the peritoneal and other serosal cavities. Definition of macrophages in bone marrow and spleen, suggested novel cell adhesive interactions and new monoclonal antibodies were subsequently isolated to discover some of the surface molecules involved; reagents for antigens such as CD169 25 exemplified and clarified the striking heterogeneity between marginal metallophils, for example, and red pulp macrophages in mouse spleen. Other monoclonal antibodies such as anti-CD68 provided pan-macrophage markers shared by macrophages 11.

**Figure 1:**
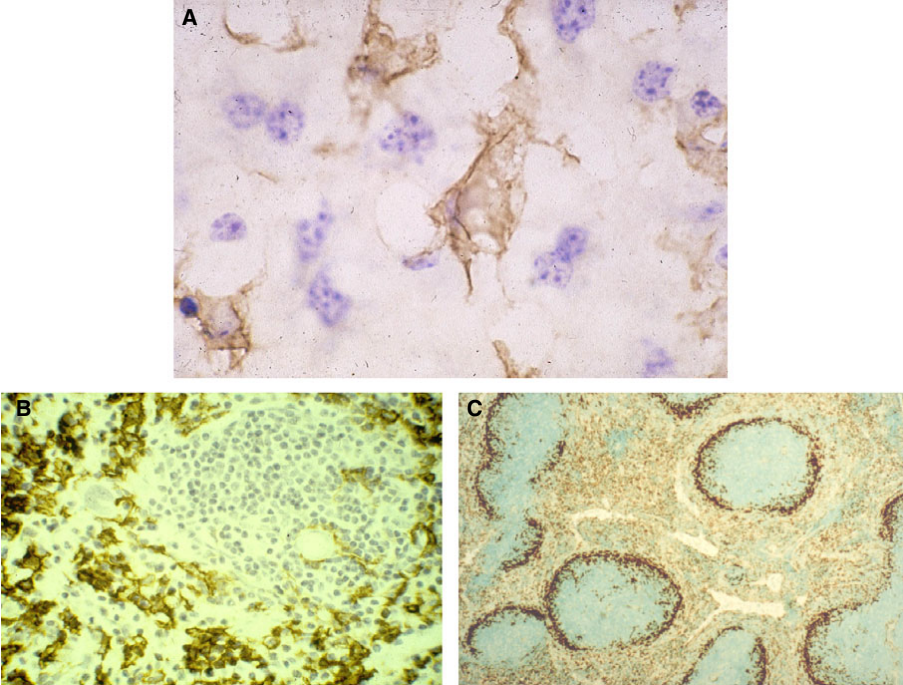
Immunocytochemistry illustrates the differential staining of macrophages in mouse liver and spleen. Kupffer cells (A) and splenic red pulp macrophages (B) are strongly F4/80^+^, unlike F4/80^−^ marginal metallophils (C), which express CD169 strongly, and red pulp macrophages, which are CD169 dim or negative. Images (A) and (B) courtesy of D. A. Hume, image (C) courtesy of P. R. Crocker.

A similar strategy was used to investigate macrophage adhesion, providing reagents to probe cell recruitment (CD11b) and scavenger receptor A (SR-A)-mediated endocytosis. Specific monoclonal antibodies were generated for previously described lectin-like receptors, such as the Mannose receptor, CD206. *Table *1 summarizes the properties and expression of a range of these and related reagents. Application of this panel of reagents revealed striking heterogeneity between different tissue macrophages, e.g. in liver and mouse spleen (*Figs *1 *and* 2), but also within individual organs such as the brain (*Fig. *3).

**Table 1:** Selected antigens expressed on murine macrophages

Ab	Ag	Structure	Ligands	Cellular Expression	Function	Comment
F4/80	F4/80 (EMR1)	EGF-TM7	?	Mature Mϕ, absent T areas; eosinophils	Peripheral tolerance	Useful marker development, microglia
FA-11	Macrosialin (CD68)	Mucin-LAMP	OX-LDL	Pan-Mϕ, DC	Late endosomal	Glycoforms regulated by inflammation and phagocytosis
5C6	CR3 (CD11b, CD18)	β2-integrin	iC3b, ICAM, *et al*.	Monocytes, Microglia, PMN, NK cells	Phagocytosis, adhesion	Important in inflammatory recruitment, PMN apoptosis
2F8	SR-A (I, II)	Collagenous, type II glycoprotein Isoforms differ, cysteine-rich domain	Polyanions, LTA; LPS; bacterial proteins, e.g. Neisseria; Modified host proteins; β-amyloid, apolipoprotein A, E	Mϕ, Sinusoidal Endothelium	Adhesion Endocytosis Phagocytosis of apoptotic cells and bacteria	Protects host against LPS-induced shock Promotes atherosclerosis Clearance of calciprotein particles
SER-4 3D6	Sn (Siglec-1)	Ig superfamily	Sialyl glycoconjugates e.g. CD43	Subsets Tissue Mϕ	Lectin	Strongly expressed in marginal zone metallophils in spleen and subcapsular sinus of lymph nodes
5D1	Macrophage mannose receptor (CD206)	C-type lectin domains (CRD) and N-terminal cysteine-rich domain (CyRD)	Mannosyl, fucosyl terminal glycoconjugates (CRD) and sulphated sugars (CyRD)	Subsets tissue macrophages	Endocytosis Adhesion	CyRD targets subsets of marginal zone metallophils in spleen.
2A11	Dectin-1	C-type lectin-like receptor hemi-ITAM	β-glucan	Myeloid cells (Mϕ, DC, PMN) ?subsets Lymphocytes	Fungal uptake	Signals via Syk and Card9

Ab, antibody; Ag, antigen; CNS, central nervous system; DC, dendritic cells; ICAM, intercellular adhesion molecule; Ig, immunoglobulin; LAMP, lysosome-associated membrane protein; LPS, lipopolysaccharide; LTA, lipoteichoic acid; Mϕ, macrophages; NK, natural killer cells; PMN, Sn, sialoadhesin; SR-A, type A scavenger receptor. For further information and references consult the text and Taylor *et al*. 11.

**Figure 2:**
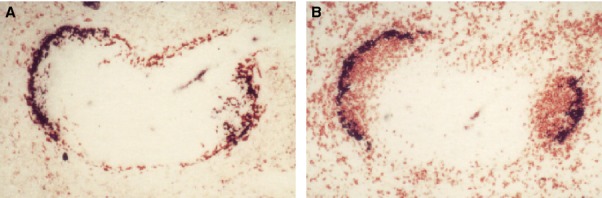
A subpopulation of CD169^+^ metallophils binds the cysteine-rich domain of the mannose receptor (CyR-Fc) (A), and associates with IgD^+^ B lymphocytes (B). Images courtesy of L. Martinez-Pomares. Reference 74 should be consulted for further details.

**Figure 3:**
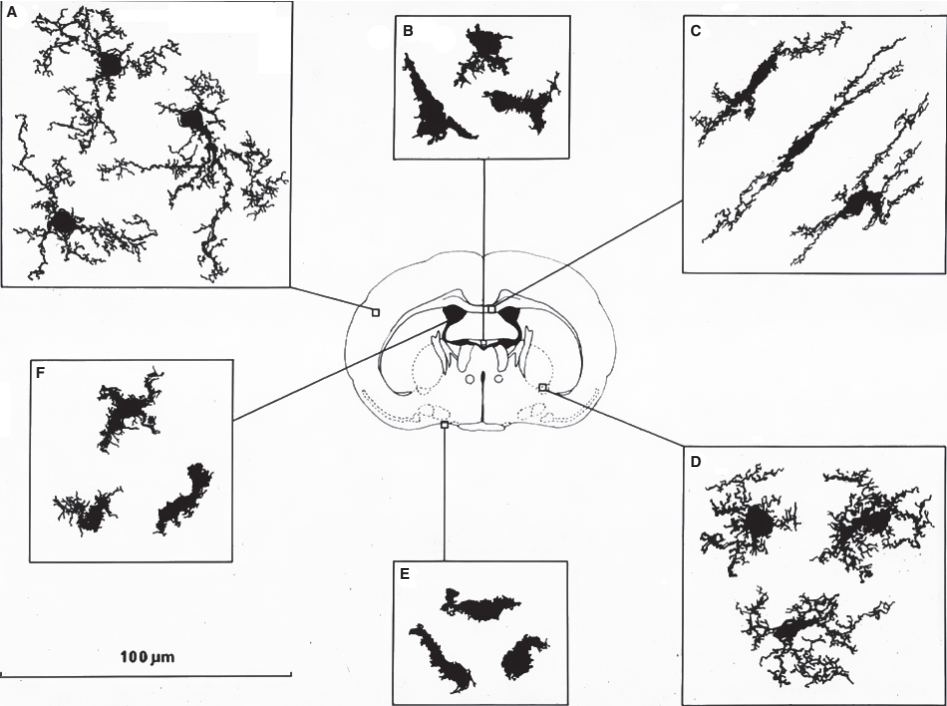
Heterogeneity of F4/80^+^ cells in adult mouse brain. F4/80^+^ microglia are present in large numbers in all major divisions of the brain but are not uniformly distributed. There is a more than fivefold variation in the density of immunostained microglial processes between different regions. More microglia are found in gray matter than white. Microglia vary in morphology depending on their location. Compact cells are rounded, sometimes with one or two short thick limbs, bearing short processes. They resemble Kupffer cells of the liver and are found exclusively in sites lacking a blood–brain barrier. Longitudinally branched cells are found in fiber tracts and possess several long processes which are usually aligned parallel to the longitudinal axis of the nerve fibers. Radially branched cells are found throughout the neuropil. They can be extremely elaborate and there is wide variation in the length and complexity of branching of the processes. The systematic variation in microglial morphology provides evidence that these cells are sensitive to their microenvironment. Drawings to illustrate the morphological heterogeneity of macrophage populations of the central nervous system: A,C,D show microglia of the brain parenchyma (A, cortex; B, white matter; C, ventral pallidum). Macrophages with a simpler morphology are also present in the circumventricular organs (B), the meninges (E), and choroid plexus (F). Based on 106 which should be consulted for further details. Image courtesy of L. J. Lawson and V. H. Perry.

Further reagents were developed by cDNA expression cloning and library subtraction. The function of Dectin-1as a β-glucan receptor was discovered by a screen using zymosan particles 26. It was then straightforward to isolate monoclonal antibodies to study tissue expression of Dectin-1 and related antigens, their regulation and signaling. In some cases, we failed to identify ligands for cloned ‘orphan’ lectin-like antigens such as MICL 27, recently achieved by others 28.

We have exploited monoclonal antibodies such as F4/80 and CD169 to study macrophage heterogeneity in wildtype and mutant mice. For example, osteopetrotic (op/op) naturally mutant mice lacking the macrophage-specific growth factor CSF-1 exhibit striking differences in F4/80+ expression: monocytes and resident peritoneal cells are markedly deficient in number as well as staining, whereas thymic macrophages are preserved; there is no expression of CD169 in op/op spleen, due to developmental absence of the marginal metallophilic population 29.

**Figure 4:**
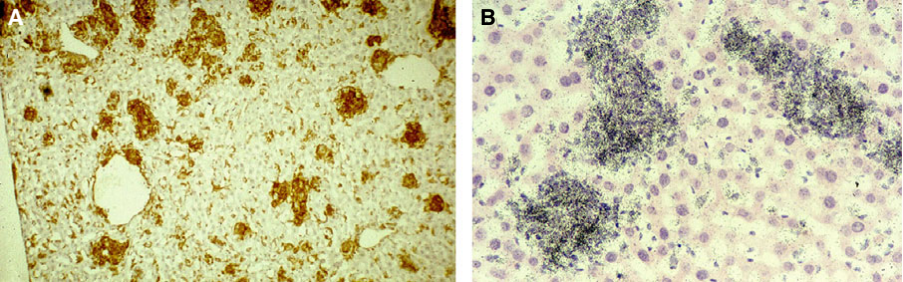
BCG-induced granulomata. BCG-induced granulomata in mouse liver express F4/80 antigen (A). These recruited macrophages are distinct in distribution from Kupffer cells and monocytes in sinusoids and express lysozyme mRNA strongly and uniformly (B). Images courtesy of D. A. Hume and S. Keshav. See Martinez-Pomares and Gordon 146 for further details.

Panels of well characterized monoclonal antibodies provide a powerful tool to analyze genetically engineered mice and human samples; however, their full potential to determine the mechanisms and significance of macrophage phenotypic diversity has not yet been realized. In this review, we summarize applications of several monoclonal antibodies produced in our laboratory over the past 3 decades (*Table *1 *and Figs *4), which helped to establish a basis for our knowledge of in situ heterogeneity of mouse macrophages in mouse tissues.

## Monocytes and macrophages in different tissues

### Embryonic and fetal hematopoiesis

Macrophages play a key role in organ development 30,31. F4/80 negative progenitors and precursors of macrophages can be detected by adoptive transfer and colony-forming assays; F4/80 staining first appears in mouse embryo at 8–9 days in the yolk sac, then in fetal liver from day 10, peaking at day 12–14. Subsequent hematopoiesis appears in bone marrow before birth. Definitive erythropoiesis is associated with the appearance of stromal-type macrophages in fetal liver, forming clusters with developing erythroblasts through non-phagocytic adhesion receptors, as discussed below. Fetal liver progenitors have been used to develop macrophage and DC lines *in vitro* 32,33. In humans, placental cord blood is also a source of progenitors. DC and macrophage differentiation diverges from earlier common precursors, through distinct transcription factors 34,35. Although they share many properties with macrophages, DCs constitute a functionally specialized, heterogeneous family of cells which are not discussed in detail in this review.

One of the key functions of yolk sac and fetal liver macrophages is to seed developing organs; recent studies have highlighted the ultimate source of the yolk sac for microglia, Langerhans cells, and many other resident tissue macrophage populations that persist throughout adult life, depending on local replenishment and continuous low turnover 4,36,37. Embryonic macrophages have been associated with phagocytosis of apoptotic cells in tissue remodeling and interaction with the extracellular matrix, important for general organogenesis and vasculogenesis 38.

Although infection is rarely surmountable in early stages of development, embryonic macrophages seem equipped with the gene repertoire to deal with pathogens 39. We know very little of the differences among different embryonic macrophage populations or how they differ from macrophages in the adult; clues may arise from studies of the changes in extracellular matrix during development, known to alter the morphology and localization of cells, as well as colonization of the gut and skin by bacteria, shaping macrophage phenotypes and interactions with other cells, and finally, increased death or proliferation of cells needed in embryogenesis for tissue remodeling. In the adult, embryonic seeded resident tissue macrophages still constitute an abundant population; postnatally, it is convenient to distinguish resident macrophages, arising during development from embryonic sources, from elicited macrophages, recruited mainly from bone marrow in response to inflammatory stimuli.

## Macrophages in lympho-hematopoietic organs

While the role of DCs as antigen-presenting cells has dominated the field, macrophages are abundant in all hematopoietic organs and influence the development of other lympho-hematopoietic cells.

### Bone marrow

Studies with monoclonal antibody F4/80 in the mouse revealed several distinct populations of mature macrophages; stromal F4/80^+^ stellate cells at the center of hematopoietic cell clusters that could be isolated by collagenase digestion of bone marrow plugs 40; F4/80^−^ osteoclasts on the medullary surface and F4/80^+^ cells at sites of muscle and tendon attachment 41. In addition, there are small numbers of F4/80 dim monocytes, which express this antigen as monocytic precursors reach a relatively late stage of differentiation. All of these cells express CD68 and Fc receptors, but other macrophage markers can vary. The stromal macrophages associate with erythroblasts and myeloblasts, while engulfing erythroid nuclei 42. CD169, a sialic acid recognition receptor, is expressed at sites of close contact with developing myeloid cells 43. This molecule has been implicated in controlling myeloid cell release from the bone marrow into the circulation 44. In addition, stromal macrophages express a divalent cation-dependent receptor (EbR), which promotes adhesion of erythroblasts and myeloblasts 45. The integrin VLA-4 and its ligand V-CAM1 may be partially responsible for these interactions; the tetraspanins CD81 and CD82 also mediate adhesion of human erythroblasts to V-CAM1 46–48. The haptoglobin-hemoglobin receptor CD163 has been implicated in iron recycling from heme, after uptake of erythrocytes 49,50, but also as an adhesion molecule for erythroblasts, suggestive of a more complex regulatory role for macrophage CD163 during erythropoiesis 51.

Although hematopoietic clusters, already described by Bessis in the 1950s 52, have also been demonstrated in human bone marrow 53, we still lack insight into the potential trophic and regulatory role of stromal macrophages in the hematopoietic niche. They are closely associated with mesenchymal fibroblastic non-hematopoietic stromal cells *in vivo*. *In vitro* studies by Allen and Dexter used dexamethasone-containing culture media and undisrupted bone marrow plugs to generate actively hematopoietic cultures which contained adipocyte-like mesenchymal cells and stromal macrophages intimately associated with developing EbR+ macrophages 54. Mesenchymal stem cells (MSCs) give rise to a variety of fibroblastic, osteogenic and adipocytic cells in culture; CD200 expression on MSC interacts with CD200R on macrophage-like precursors, inhibiting osteoclast formation 55. Stromal macrophages from NOD mice express a polymorphic SIRP α variant which through binding of the ‘don't eat me’ CD47 molecule, limits phagocytic clearance and improves engraftment of such mice by human hematopoietic stem cells 56,57.

Aside from stromal macrophages in hematopoietic clusters, another important bone marrow macrophage-related cell is the osteoclast. Osteoclasts are multinucleated giant cells that arise by fusion of monocyte-like cells in the presence of CSF-1 and RANK ligand 58. Their localization is not restricted to the bone marrow niche but extends to the whole bone. Cell surface molecules such as DC-STAMP have been implicated in osteoclast fusion; multiple transcription factors including NF-κB, c-Fos, and NFATc1 regulate this process. These cells, which collaborate with osteoblasts in bone production and remodeling, are uniquely able to resorb living bone, by tight integrin-dependent adhesion to the bone surface, expression of carbonic anhydrase, and polarized secretion of HCl and proteolytic enzymes. In addition to their conventional role in bone resorption, osteoclasts regulate the differentiation of osteoblast precursors, the movement of hematopoietic stem cells from the bone marrow to the bloodstream and overall immune response 59.

We do not know how these distinct mature macrophage populations arise within the specialized environment of bone; fetal liver macrophages share some of the properties of stromal macrophages, expressing mainly the divalent cation-dependent receptor for erythroblast adhesion. Similar cells are found in splenic red pulp.

### Blood

Circulating blood monocytes are the predominant macrophage family cells in blood. Monocytes can interact directly with endothelia to perform poorly defined housekeeping functions 60 or, upon activation, enter tissues at local sites of injury, inflammation, and infection. Monocytes are profoundly influenced by their microenvironment and, in turn, affect endothelium and extravascular tissue integrity. Here we consider some of the principles that underlie their interaction with other blood constituents, as a preliminary to dealing with specialized aspects in tissues, such as granuloma formation.

Because of their ready availability, much of our knowledge is derived from studies with human monocytes *ex vivo*, but work in the mouse has provided important information of their origin and distribution *in vivo*. Monocyte heterogeneity is well established in both species, based on expression of chemokine receptors and panels of surface antigens such as CD14 and CD16 for human 61–63 and Ly6C, CCR2, and CX3CR1 in mouse 64,65. Some phenotypic similarities have been described across species 66. The morphology of monocytes has been well documented 67; they contain rudimentary secretory granules containing lysozyme and myeloperoxidase, in comparison with polymorphonuclear leukocytes.

The nature of monocyte heterogeneity is not always clear, as more markers are found to be expressed on subpopulations, which do not necessarily correspond to subsets defined by commonly used markers. Traditional subsets can also change in response to physiologic influences such as exercise, as well as disease 68. Monocytes change their phenotype upon adhesion to endothelium and diapedesis, although some cells retain a monocyte character within tissues 69 and some tissue macrophages may re-enter the circulation. Activated monocytes affect endothelial cell permeability as well as angiogenesis.

Monocytes interact potently with plasma proteolytic cascades (coagulation, fibrinolysis, complement, kinins), contributing and responding to cascade activation, through secretory product generation and receptor expression, extracellular proteinase activation and inhibition 2. They can interact explosively with activated platelets and polymorphonuclear leukocytes, form adherent aggregates, and migrate readily in response to chemokine stimulation. Platelets are potent sources of procoagulants, lipid mediators and growth factors, as well as TGF β, relevant to macrophage function in tissue repair. Activated neutrophils and monocytes themselves generate potent oxygen-derived radicals required for host defense, but are also responsible for tissue injury. Lymphoid cells and their products are important in chronic inflammation, cell-mediated immunity and delayed type hypersensitivity, whereas erythrocytes provide a sink for complement-derived peptides.

The contribution of monocytes to disease processes such as atherogenesis is critical to pathogenesis of arterial occlusion, thrombosis and plaque rupture 70–72. Other intravascular consequences of infection and trauma include Dengue virus–induced hemorrhagic shock 73. While the functions of activated monocytes are well described, we are left with uncertainty regarding monocyte functions in the steady state, the effects on circulating monocytes of peripheral injury, and the response of monocytes to hemodynamic perturbations.

### Spleen

Spleen is a hematopoietic organ that provides a striking example of phenotypic diversity of macrophage-like cells. These have been well described in rodents by Kraal, Mebius, and Martinez-Pomares *et al*. 74,75. Some of the marker properties of red and white pulp and the intervening marginal zone (sinus-lining metallophils and outer marginal zone) are listed in *Table *1. Reflecting their major function in clearance of senescent erythrocytes, a subset of F4/80^+^ red pulp macrophages respond to heme by induction of a specific transcription factor, Spi-C and express properties associated with heme catabolism and iron storage and reutilization 76. White pulp macrophages express CD68 in abundance, but lack F4/80; the cDC migrate readily into white pulp following antigen challenge and are mainly responsible for activation of T lymphocytes 77.

The F4/80 negative marginal zone is at the interface of the circulation and resident lymphoid cells; metallophils express CD169, whereas the outer more phagocytic marginal zone cells express DC-SIGN/SIGNR1 and the Scavenger Receptor MARCO. These cells capture microbial polysaccharides and also clear calciprotein particles 78. They play an important role in viral infection, and in transport of microbial antigens to B and T cells.

The mannose receptor (CD206), which is strongly expressed in red pulp, plays a complex role in splenic macrophage functions; it is a clearance receptor for mannose-, fucose- and GlcNAc–terminal glycoconjugates and microbial ligands 79. Additionally, its N-terminal cysteine-rich domain binds sulphated sugar ligands in a subset of CD169^+^ metallophils, providing a double lectin bridge, to promote clearance. The MR has an additional fibronectin-like domain implicated in matrix binding. Not only mature macrophages populate the spleen; Swirski *et al*. 80 have demonstrated that undifferentiated monocytes reside in the spleen, outnumber their equivalents in circulation, and are recruited from spleen to sites of inflammation such as myocardial infarction. Further work is needed to understand the heterogeneity of macrophages in human spleen, which remains poorly defined.

### Lymphoid tissues

As in spleen there is remarkable macrophage heterogeneity in lymphatic organs 74. Compared with spleen, the F4/80 antigen is less marked in expression, whereas CD169 is a prominent marker of specialized macrophages lining the subcapsular sinus, analogous to marginal metallophilic macrophages. Afferent lymph contains tissue macrophages and CD11c^+^ DCs laden with potential antigens. Lymphatic vessels also express the mannose receptor, CD206. The fate of antigen capture by antigen-presenting cells (APCs) and delivery to B and T lymphocytes has been well described by Jason Cyster, Ron Germain, and their collaborators 81,82. Viruses and immune complexes have been used to study innate and adaptive lymphoid cell interactions in lymph nodes. Lymph is collected by reticular channels and sinuses and exits from the medulla. The medullary cords are rich in F4/80^+^ macrophages and plasma cells. Lymph depleted of macrophages leaves via efferent lymphatics. The stroma of lymph nodes and local cell adhesion interactions mediated by podoplanin have been studied by Shannon Turley *et al*. 83, but we lack information as to how distinct phenotypes of macrophage populations are regulated. Cytokine and growth factor–deficient mice have revealed how the anatomical compartments arise during development. F4/80 is also downregulated in lymphoid-rich Peyer's patches in the intestine.

The macrophage population of the thymus 84 has been neglected, compared with DCs and specialized epithelial cells involved in thymocyte selection; efficient phagocytic clearance of apoptotic thymocytes and hydrolysis of DNA in apoptotic cells upon their phagocytosis of the dead cells is a major function of macrophages. The heterogeneity of these cells has begun to emerge but needs further studies 85,86.

## Macrophages in liver

Considering their prominent contribution to the RES, the resident macrophages of the liver, cells identified by Von Kupffer and characterized by Browicz as macrophages, have recently been relatively neglected 87. They constitute an apparently homogeneous abundant population of sinusoidal clearance cells, in direct contact with the portal circulation and adjacent hepatocytes. They therefore deal with gut-derived products and potential toxins, yet are able to influence the synthetic and metabolic powerhouse of the body, and are critical for physiologic homeostasis as well as providing portals for infection by hepatitis virus and malaria parasites. They contribute to iron storage, lipid dysregulation (steatosis), liver repair, fibrosis and cirrhosis following alcoholic hepatotoxicity. Their numbers can be augmented by recruitment of newly recruited monocytes/macrophages, with distinct antimicrobial and inflammatory capacity, compared with resident Kupffer cells, presumably tolerized by lipopolysaccharide exposure 88.

Kupffer cells, strongly F4/80^+^, can be distinguished from F4/80^−^ true endothelial sinusoidal cells and from F4/80^+^ infiltrating interstitial monocytes and macrophages. They express low levels of CD11b, the type complement receptor, but express a unique Ig-complement receptor (CRIg) 89. Together with sinusoidal F4/80^−^ endothelial cells, they express selected FcR, several lectins, especially CD206, and Class A scavenger receptor. In rat liver, Kupffer cells express a distinct tissue-specific fucose receptor. Perhaps because of cell extraction difficulties, and because of rapid changes in phenotype following isolation, we do not have sufficient knowledge of Kupffer cell gene expression *in situ*.

Their origin during fetal development is initiated during the transition from primitive to definitive erythropoiesis, which declines in the liver postnatally; as the bone marrow matures, the stromal-type macrophages described above seem to transform into resident sinus-lining cells, but the origin of the subsequent major population of Kupffer cells in the uninfected host, deserves further investigation, given earlier claims of a high level of recruitment from circulating monocytes 90.

Macrophages at local sites are able to produce low levels of many plasma proteins, including all components of the complement pathway, mimicking the far higher levels produced by hepatocytes 2. They can even produce bilirubin. The expression of clearance receptors by both Kupffer cells and sinusoidal endothelium suggests common regulation by a specialized local milieu. Given the role of selective transcription factors in tissue–specific red pulp spleen macrophages (Spi-C) and of Gata6 in peritoneal cells, discussed below, one might predict a similar tissue-restricted transcription factor for Kupffer cells. Since retinoids play an important role in the latter case, the presence of vitamin A-storing hepatic stellate in close vicinity in the space of Disse may also be relevant.

In spite of these considerations, the interactions between hepatocytes and macrophages in the liver are under-investigated. This may change as improved hepatocyte cell culture models become available.

## Macrophages in gastrointestinal tract

### Gut macrophages

The gut represents the most abundant source of resident tissue macrophages 41,91; F4/80^+^ cells are present in the *lamina propria* throughout its length, even in the fetus. Although these cells do not necessarily send projections into the lumen of the intestine, as do DCs, they may be tolerized by their exposure to microbial flora. Any breach in epithelium also exposes macrophages to luminal contents. The origin and presence of macrophage subpopulations have been extensively documented, as have DC subpopulations, both in healthy subjects and in inflammatory bowel disease 92–94. TGF β, presumably stromal in origin, and IL-10 have been implicated in the phenotype of *lamina propria* macrophages. However, the presence of abundant innate and adaptive lymphocytes, ILC2, NK and NKT, as well as B cells, αβ and γδ T lymphocytes, mast cells and other myeloid cells, let alone a multiplicity of microorganisms, all provide a rich mix of products able to modulate the phenotype of gut macrophages. Studies with germ-free, co-housed, as well as genetically modified mice followed by *in situ* and *ex vivo* macrophages can now be done in isolated as well as co-culture models.

### Peritoneal macrophages

Much of our knowledge of the *ex vivo* properties of primary macrophages in the mouse derives from washout of the peritoneal cavity of unstimulated animals (resident), after injection 3–4 days prior to harvest of inflammatory agents such as thioglycollate broth, zymosan particles, or biogel polyacrylamide beads (elicited) and of BCG/C Parvum (activated macrophages) 2. The phenotypes of the macrophages differ markedly in antigen expression, adhesion, phagocytic and secretory functions, including less frequently studied characteristics such as the ability to fuse following IL-4 exposure in culture 58. Resident macrophages are strongly F4/80^+^, F4/80 expression levels of elicited peritoneal macrophages drop after influx of monocytes, before recovery of high levels in culture. The peritoneal cavity is not a closed system, but the population changes due to influx, emigration to draining lymph nodes, adhesion to mesentery and cell death/proliferation. Milk spots contain F4/80^+^ macrophages adherent to mesentery. All these macrophages, and other lymphoid and myeloid cells, are exposed to microbial constituents that pass across the gut wall. These include TLR agonists, which induce plasma membrane receptors such as MARCO (S. Mukhopadhyay, unpublished observation).

Although peritoneal macrophages have been extensively characterized by FACS and microarray analysis, studies by Taylor, Medzhitov, Randolph *et al*. 95–97 have only recently identified Gata-6 as an important peritoneal–restricted transcription factor, associated with proliferative potential. Retinoids have been shown to regulate peritoneal cellular responses.

The peritoneal cavity facilitates cell recovery without digestion at different times after administration of defined antigens and can be exploited to examine infection, cell transfer and tumorigenicity, but needs to be used with appropriate care to control for cell recovery and for individual cell heterogeneity.

## Macrophages in the lung

The macrophage populations of the lung display striking variation, depending on access to the airway by alveolar macrophages, which encounter pollutants and particulates that reach the lower airway, and interstitial macrophages, not directly exposed to exogenous stimuli. High oxygen levels in the microenvironment may contribute to oxidative injury. Local products such as surfactant proteins are also potential opsonins and macrophage modifying factors. Further specialization includes the arborized intra-epithelial cells, both macrophages and DCs, in larger airways. Finally, there are macrophages in the pleural cavity, which are less studied than serosal cells in the peritoneal cavity.

Alveolar macrophages have a distinctive rounded morphology and can be isolated from mice as resident and elicited cells, following exposure to irritants, allergens or infectious agents. These macrophages have been known to be self-renewing populations since the introduction of bone-seeking isotopes and studies with parabiotic mice. Recent studies by Guilliams *et al*. 37 have shown that alveolar macrophages develop from fetal monocytes that differentiate into long-lived cells in the first week of life via GM-CSF.

Alveolar macrophages express low levels of F4/80 and of CD11b. GM-CSF deficiency is associated with failure of surfactant clearance, resulting in alveolar proteinosis. Alveolar macrophages express high levels of lectins such as CD206, and of Scavenger Receptor AI/II and MARCO, induced by, and involved in, uptake of inhaled particles. These receptors are important in host defense against a wide range of airborne microorganisms, especially in the absence of opsonic antibodies and sufficient complement.

The group of Tracey Hussell 98 has studied acute co-infection by viral and Gram-positive bacterial pathogens via the airway, as well as the resolution of pathologic lesions after inflammation. A range of molecules has been identified at different stages of the disease process, which does not fully restore the pristine state of the airway with time.

Asthma, which occurs as allergic or non-allergic conditions, gives rise to epithelial injury, inflammatory and immune cell infiltration and activation, including monocytes and macrophages, as well as bronchial smooth muscle and goblet cell hyperplasia, and enhanced mucus production. Th2 cytokines, IL-4/13 and IL-5, which act on macrophages and eosinophils, contribute to allergic pathology, impaired tissue remodeling, and fibrosis. Other fibrotic lung disease processes in which macrophages play a major role include silicosis and other pneumoconioses, and asbestosis. The nature of the inhaled particulates, especially their ability to activate complement and their degradability, contribute greatly to chronic inflammation, concomitant tissue destruction and resultant malignancy. Mycobacterial infection has taught us a great deal about macrophage heterogeneity in tuberculous pathology, including granuloma formation, considered below.

## Macrophages in the skin

Malissen *et al*. 99 have recently defined heterogeneity in various macrophage and DC subpopulations in skin. Langerhans cells in the epidermis are morphologically distinct, F4/80^+^ specialized macrophage-like cells 23 that originate from yolk sac precursors 100,101 and persist through adult life in close contact with clusters of keratinocytes, reciprocally exchanging survival and trophic factors. They can be induced by antigen stimulation and TNF to migrate to draining lymph nodes where they are able to activate or inhibit lymphocytes. Birbeck granule formation is characteristic of a specialized antigen-processing compartment and Langerin (CD207) is a useful marker for Langerhans cell histiocytosis 102.

Mutations in the human keratinocyte-expressed filaggrin gene are relatively common and associated with an increased frequency of Th2-dependent allergic conditions such as atopic dermatitis 103. The role of Langerhans cells in initiating allergic responses in this condition merits further investigation.

Dermal F4/80^+^ macrophages are associated with adnexal structures such as hair follicles and resemble other macrophages in connective tissue.

## Macrophages in the nervous system

Early staining observations suggested that ramified macrophage-like cells, named microglia by Pio del Rio Hortega in the 1920s, performed phagocytic housekeeping functions in normal and diseased brain 30,104,105. Bone marrow transplantation indicated a hematopoietic origin and immunohistochemistry for markers such as F4/80 and Iba1 revealed their widespread distribution and heterogeneity within the central nervous system 106. Yolk sac-derived precursors 4,107,108 enter the brain during development at a time of extensive neuronal apoptosis and subsequent clearance by macrophages, and persist as resident microglia, which turn over slowly throughout adult life; microglia are strikingly activated in response to local injury and excitotoxic stimulation, and their numbers are supplemented by recruited inflammatory monocytes/macrophages of bone marrow origin 105.

Morphology and antigen marker analysis show that microglia adopt a highly heterogeneous regional phenotype within their unique microenvironment, illustrated by the F4/80 antigen, which is well expressed on microglial plasma membrane processes 106 (*Fig. *3). Microglia are unusual compared with other resident macrophage population outside the brain, in their constitutive expression of CD11b, the type 3 Complement receptor. This molecule has been implicated by confocal imaging in synaptic pruning during development and postnatally 109,110. Other molecules implicated in microglial phagocytosis of neurons are UDP/P2Y6 111 and Lactadherin/MFG-8 112.

The F4/80 antibody also labels perivascular, choroid plexus 113 and leptomeningeal macrophages, different in appearance from ramified microglia. Perivascular macrophages, for example, express CD206 and SR-A, unlike resident microglia, consistent with clearance functions. Expression of CD169 in resting conditions, is restricted to circumventricular organs outside the blood–brain barrier, suggestive of a humoral inducer 114.

The primary role of microglia in removal and digestion of naturally dying neurons and effete synaptic constituents, as a result of overproduction or lack of functional stimulation, is likely to be mediated by the balance between a range of apoptotic recognition and ‘don't eat me’ signals; the receptors and ligands need further definition. Remarkably, microglia seem able to ‘nibble’ plasma membrane and cellular components by intimate contact with live neurons, demonstrated, for example, in the uptake of neurohypophyseal granule hormones into their lysosomes 115.

Synaptic trimming continues throughout adult life and clearance of extracellular plaques, tangles and aggregates of neuronal origin are significant factors in neurodegeneration and ageing.

The role of resting and activated microglia in glucose, neurotransmitter and glutamine metabolism 116 and hypoxia, as well as infection, neuroinflammation, neurodegeneration, and gliosis deserve further study. Systemic and local cytokines such as IL-1, TNF and IL-4 have been implicated in fever, anorexia, and cognitive dysfunction.

Myelin is exquisitely sensitive to macrophage-derived proteases. The kinetics of Wallerian degeneration of peripheral nerve after transection, in which macrophages play an important role, was found to depend on a neuronal expressed gene, wld 117,118. Macrophages of different types (monocytes, microglia, choroid plexus macrophages) play a substantial part in spinal cord injury and repair 119. Their possible role in pain of neuropathic and inflammatory origin deserves further study.

The anterior chamber of the eye is an immunologically privileged site; the F4/80 antigen has been implicated in a peripheral tolerance model (ACAID) 120. TGF β, IL-10 and other deactivating local environmental signals have been identified.

The signals that control macrophage responses within the unique microenvironment of the central and peripheral nervous system provide a fertile field for further investigation. Advances in intravital imaging and *ex vivo* brain slice analysis provide promising approaches to cross the divide between the intact organism and isolated cells.

## Macrophages in endocrine organs

Macrophages are present in all endocrine organs constitutively and monocytes can be recruited to pancreatic islets, for example, by autoimmune and other inflammatory stimuli. Their distinction from DCs has been confused, due to inappropriate use of single markers such as CD11c. A subpopulation of cDC express F4/80, but this is typically a marker of macrophages. Resident F4/80^+^ macrophages are present in pancreas 121, thyroid, adrenal gland, and anterior and posterior pituitary 106,122. Sinusoidal macrophages in endocrine tissues as well as liver (Kupffer cells), influence circulating levels of hormone; leutropin is cleared via recognition of sulphated sugars by the N-terminal cysteine-rich domain, whereas thyroglobulin is a ligand for mannosyl-glycans by CD206 123. Enzymatic activities of adrenal macrophages regulate the metabolism of glucocorticosteroids, which in turn exert potent effects on cytoplasmic and nuclear receptors of monocyte/macrophages themselves. Macrophage functions contribute to homeostasis, as well as dysfunctions associated with metabolic disease, inflammation and infection.

## Macrophages in the cardiovascular system

### Heart

The normal heart contains stellate macrophages between muscle fibers and cells beneath and within the pericardium; myocardial infarction results in acute and chronic inflammatory recruitment 124. Genetic fate mapping revealed that yolk sac and fetal monocyte progenitors give rise to a substantial fraction of cardiac macrophages that persist in adulthood 125. In the steady state, resident macrophages are maintained through local proliferation of these embryonically established resident macrophages. After macrophage depletion or during inflammation, resident macrophages expand considerably through proliferation, while a wave of Ly6c^Hi^ blood monocytes enter the tissue. At least four macrophage populations have been identified in the heart based on the expression of Ly6c, CD64, MERTK, MHC-II, CD206, and CD11c.

CCR2 expression and dependence distinguished cardiac macrophages of adult monocyte versus embryonic origin. Heart macrophages participate in clearance of apoptotic cardiomyocytes, vasculogenesis as well as angiogenesis during development, coordination of inflammation, repair and neoplasia. In humans, little is known about cardiac macrophage phenotype aside from CD68^+^ expression and overall localization.

### Vasculature macrophages

Here we draw attention to macrophage recruitment to arteries during atherosclerosis and its complications, plaque rupture, and thrombo-embolism. Uptake of modified lipoproteins and cholesterol by scavenger receptors results in foam cell formation and a distinctive phenotype of gene expression, including enzymes involved in lipid metabolism, secretion of procoagulants and metalloproteinases 126. CD68 antigen is strikingly enhanced by lipid uptake whereas CD163 expression is a marker of macrophage heterogeneity. CD163, a receptor for hemoglobin-haptoglobin complexes, is implicated in heme catabolism, e.g. after intraplaque hemorrhage 49. Finally, angiotensin converting enzyme expression by macrophages indicates a role in hypertension and is a marker of macrophage activation in granuloma formation, as in Sarcoidosis.

### Macrophages in adipose tissue

Recent studies have emphasized the proinflammatory phenotype of macrophages in adipose tissue, during obesity, compared with lean mass 127. Interactions between adipocytes and macrophages play a reciprocal role in metabolism in part through PPAR transcription factors 128.

### Macrophages in kidney

The F4/80 antigen is present on interstitial resident macrophages, but is strikingly absent in normal mouse glomerular tuft vessels and mesangium 129. However, F4/80^+^ macrophages line Bowman's capsule, and are concentrated near the juxtaglomerular complex, a site of renin processing and erythropoietin production. Immune complex deposition and other inflammatory conditions induce glomerular infiltration by macrophages and other leukocytes; aside from FcR, mesangial CD206 has been shown to be essential for proteinuria in mouse models of immune complex glomerulonephritis 130.

### Macrophages in muscle and connective tissue

F4/80^+^ macrophages are present between fibers in normal skeletal muscle and throughout connective tissue 41, but recruited monocyte/macrophages become prominent as a result of muscle cell injury and death, playing a role in removal of debris and fibrosis, for example in the mdx dystrophy model; IL-10 enhances macrophage promotion of satellite cell proliferation 131,132. AMPK α1 has been implicated in skewing of the macrophage phenotype in muscle regeneration 133,134.

### Macrophages in reproductive organs

Macrophages contribute to uptake of apoptotic sperm in the testis, an immunologically privileged organ. F4/80^+^ macrophages constitute approximately 20% of testicular interstitial cells 122. A dense network of DCs has been observed in mouse epididymis, and may play a role in immunological tolerance and auto-immunity 135.

In the ovary and uterus, macrophages fluctuate in the course of the normal menstrual cycle. Recruitment of F4/80^+^ macrophages is a striking feature of luteolysis and follicular atresia 122.

Brandon 136 used F4/80 and CD68 staining to study the role of macrophages in involution of the mouse uterus following parturition. The phenotype of macrophages in the non-pregnant uterus has been documented by Hunt *et al*. 137. Pollard *et al*. 138 investigated the pregnancy defect in the osteopetrotic op/op mouse, demonstrating a requirement for CSF-1 in female fertility. Uterine epithelium, a source of CSF-1, is associated with recruitment of SR-A^+^ macrophages, thought to contribute to growth and remodeling during the menstrual cycle (J. Brandon, unpublished observations). Endometriosis is a poorly understood condition where ectopic epithelial cells induce peritoneal inflammation, associated with a distinct pro-inflammatory macrophage population, revealed by network analysis 139.

### Decidual macrophages

As a result of pregnancy, there is an expansion of decidual macrophages, probably derived from maternal monocytes, at the interface with syncytiotrophoblast, which, in human pregnancy, invades deeply towards spiral arteries to access maternal blood and nutrients 140. Decidual macrophages are also intimately associated and likely to interact with NK cells (non-cytotoxic in this environment), possibly contributing to immune tolerance of alloantigens. They express receptors for CSF-1, GM-CSF, and IL-10, all three products of NK cells. The more abundant macrophage-like cells express CD14, HLA-DR, CD11c, and DC-SIGN, but there is marker overlap with other APC, a minor population of CD14 negative DCs. Other markers include CXCR1, I-CAM-1, and LILBR1 (leukocyte immunoglobulin-like receptor B1), which binds HLA-G homodimers on trophoblasts. Decidual macrophages express low levels of costimulatory molecules (CD80, CD86), which may be upregulated after maturation stimuli, acquiring the ability to activate T lymphocytes. Treg activation has also been described. The scavenger receptor stabilin-1 is a receptor for placental lactogen 141.

Decidual macrophages have been extracted from human placenta and subjected to microarray analysis, compared with blood monocytes and, to a lesser extent, with other tissue macrophages (see below) 142,143. The cells are remarkably plastic and express a range of both M2 and M1-associated genes, but do not fit a simple bipolar classification. Further studies are required to establish subpopulation diversity and the influence of hormonal and neighboring cells in a unique environment. In this regard, their ability to produce high levels of collagens, laminin and extracellular matrix are of particular interest. The role of decidual macrophages in disorders of early pregnancy deserves further investigation.

### Hofbauer cells

Hofbauer cells are fetal macrophages found in developing villi of the placenta from day 11 in mouse and day 18 in human, persisting till term, although more difficult to detect at later stages of pregnancy for technical rather than biological reasons 144–146. They are large, 10-30 microns, often vacuolated and granular in appearance, consistent with active endocytosis and phagocytosis. Their fetal origin, shown by Y-chromosome markers in human, is distinct from that of maternal decidual macrophages; in the mouse, studies with the F4/80 antigen are consistent with a yolk sac origin. They express general properties of tissue macrophages, e.g. CD68, lysozyme, acid phosphatase, Class II MHC, CD206 and lectin (Maclura pomifera) binding. They show features consistent with stimulation, e.g. induction of CD163 by glucocorticoid treatment in prematurity, and express high levels of peroxiredoxin 5 and IDO. Existence of subsets requires further definition; plasticity, perhaps associated with their unique dynamic hormonal environment, has complicated classification as M2-like in phenotype. Microarray analysis of isolated Hofbauer cells indicates striking differences in the macrophage expressed gene signature; further studies are needed to control for the duration of pregnancy, termination circumstances and validation of proteins.

Their specialized functions are unproven, but suggestive; these include development and remodeling of vascular and mesenchymal elements in villi, promoting fusion of syncytiotrophoblast, branching morphogenesis of the placental villous tree, immunological tolerance and defense against infection across the maternal–fetal interface. Their contribution to villous inflammatory conditions and complications of pregnancy is unclear.

## Macrophages in granulomata

One of the questions raised earlier relates to the impact of different tissue environments on monocytes and macrophages recruited as a result of inflammation and infection. Even in highly specialized tissues, for example brain, it can be difficult to distinguish these activated cells from resident macrophages such as microglia, considered above. Granulomata provide useful models to explore this issue. *Fig. *4*A* illustrates F4/80 expression in BCG-induced granulomata in mouse liver, which provides a relatively uniform background of Kupffer cell staining; *Fig. *4*B* demonstrates lysozyme mRNA expression by the bulk population of activated macrophages, detected by *in situ* hybridization 147. Expression of TNFα message, by contrast, is restricted to a subpopulation of activated macrophages (data not shown).

Granuloma formation involves dynamic 148, organized collections of recruited monocytes/macrophages, together with lymphoid and other myeloid cells, especially eosinophils and neutrophils, with connective tissue cells, particularly fibroblasts and vascular/lymphatic elements. These are often associated with chronic inflammation and giant cell formation but include foreign body granulomata. It is customary to distinguish Th1 type, e.g. in tuberculosis, and Th2 type granulomata, e.g. surrounding *Schistosome* eggs 149. Granuloma formation is exceptional in viral infection but can accompany autoimmune/idiopathic inflammatory disorders such as sarcoidosis, Crohn's disease, and Wegener's granulomatosis. Granulomata can be relatively benign or tissue destructive, e.g. in bone, but are not malignant, monoclonal disorders except where mimicked by lymphoid malignant diseases such as Hodgkin's disease.

The foreign body type granuloma tends to be relatively inert, without lymphoid cell activation; immune granulomata cells turn over more actively, and cytokines such as TNF and IL-4/13 play an important role in their formation 149,150. The macrophage phenotype depends on the nature of the eliciting agent, e.g. persistent infection by poorly degradable mycobacteria or parasite eggs, as well as the cytokine environment. In tuberculosis, the mycobacterial lipids contribute to giant cell formation and macrophage fusion. The lesions contain recently recruited CR3^+^ monocytes, so-called epithelioid cells 151, unfused macrophages, and polykaryons (Langhans giant cells) 152,153. Differences among these macrophage populations have been described, with regard to antigen expression, endocytosis/phagocytic and secretory activity, but need further validation *in vivo*. Th2 type multinucleated giant cells, dependent on fusogenic IL-4/IL-13, lack bone degradation capacity, demonstrated by osteoclasts, with which they have much in common (Helming, Milde, unpublished observations).

It is not clear whether particulates, T cells/macrophages or stromal cells/extracellular matrix are the primary determinants of the macrophage phenotype in granuloma formation and fibrosis. It would be interesting to compare the phenotypes of granuloma macrophages with those of foam cells in atherosclerosis and with tumor-associated macrophages in malignant diseases. Such studies would provide insights into the importance of environment in macrophage heterogeneity during granuloma formation, the possible advantage to host or pathogen of sequestration, the relevance to restricted cellular migration or retention in tissues, and metastasis.

## Tissue macrophages in the OMICS era: need for integration

Although we have learned a great deal about macrophages, our understanding is still fragmentary, and an overall view of tissue-specific functions, selective markers, or even accurate numbers of macrophage subpopulations in health and disease is lacking, especially in humans. Examples and fragmentary evidence are becoming available, but tools need to be applied in a consistent manner to gather a general perspective of the macrophage system and its functional heterogeneity. Genomics, transcriptomics and proteomics have advanced our understanding of the complexity of macrophage gene signatures and their phenotypic modulation during physiologic and immune responses. *In vitro* and *ex vivo* studies have shown that macrophages respond with consistent gene programs that behave in a modular fashion 154–156; these modules can be regulated by many extrinsic tissue environmental factors including cytokines, cell to cell contact, and the extracellular matrix, but also usefully as well as detrimentally by drugs. Although at single gene level there is little specificity, this can be achieved increasingly by studying gene signatures as a whole. Importantly, these signatures are not fixed, but change in response to the same stimulus over time, as well as tissue localization. As we aim to understand the genome-transcriptome-proteome-interactome-phenome relationship for macrophages in every tissue and in different diseases, integration of all these analyses poses a formidable challenge.

Detailed studies of tissue macrophage gene expression and their heterogeneity within and among many tissues are still limited, especially in humans. Gautier, Randolph *et al*. reported in 2012 157, as part of the Immgen project, on the identity and differences in mouse tissue macrophages; four populations were assessed as a whole, peritoneal, red pulp splenic, lung macrophages, and microglia. Recently, Okabe and Medzhitov 97 assessed macrophages from peritoneal cavity, lung, liver, spleen, intestine, and adipose tissue. Candidate tissue-specific overexpressed markers from these studies included F5, FN1, and CD102 for peritoneal macrophages, Ear2, Ear3, Chi3L4, and chi3l3 in alveolar macrophages, CD207, GPR182, CD26, and clec4g for liver macrophages, CD121b, MMP2, and MMP13 for gut macrophages and CD82, SPIC, and CCR3 for spleen. When comparing the gene lists for specific tissue macrophages reported by both studies, e.g. peritoneal macrophages or spleen macrophages, we find similarities and differences that need further investigation. This is almost a hallmark of microarray studies so far; isolation procedures, array platforms, inter-individual, strain and housing differences, among other factors, are all able to affect gene signatures. In humans, similar variability has been shown for monocytes, for example 61. Proteomics and phenomics assessment have not usually been carried out at this level of analysis.

An important ultimate goal is to understand the modulation and interpretation of macrophage signatures in a broad range of disease states. Alveolar macrophages provide one of the best-studied populations in both species; we focus in the following paragraph on lung because findings in this system carry lessons that can be applied to macrophages in other locations. The lung is an exceptional organ for phenomic studies because of the highly developed tools to assess organ performance. In lung, Shaykhiev, Crystal *et al*. 158 defined gene expression signatures in smokers versus non-smokers and chronic obstructive pulmonary disease (COPD) patients to detect a large number of expressed mRNAs and signs of repression of interferon signatures and exacerbation of trophic signatures in smoking and more COPD patients. This study shows a relation between disease cause and consequence based on gene signatures. Furthermore, it reveals that the genes that we find to be important for activation *in vitro* appear as modules *in vivo*, but with unexpected tendencies; for example macrophages from healthy individuals have basally more Interferon genes expressed than smokers or COPD patients. It does not necessarily mean that alveolar macrophages are constitutively activated. Interpretation of *in vitro* findings in relation to *ex vivo* data can be difficult, because strong markers of IL-4 and steroid signaling such as MRC1 and CD163 are almost ubiquitously expressed in all tissue macrophages, requiring further investigation (FME, personal observation).

An elegant demonstration of how the cellular environment affects the alveolar macrophage response was published by Snelgrove, Hussell *et al*. 159. They showed that airway macrophages have high expression of the negative regulator CD200 receptor (CD200R) and their inflammatory potential was restrained by CD200 expressed on airway epithelium. Mice lacking CD200 had more macrophage activity and enhanced sensitivity to influenza infection and consequent mortality. How specific cellular, matrix, and soluble signals influence macrophage responses need to be investigated in detail.

Infection is also a strong modulator of tissue macrophage activation and development of specialized phenotypes. An interesting study addressed inflammatory signatures in laser micro-dissected macrophages in caseous human pulmonary tuberculosis (TB) granulomas 160. Microarray and histology confirmed overrepresentation of lipid sequestration and metabolism signatures in granuloma macrophages. Phenotypically, this could be relevant to pathogenesis since the caseum has an abundance of cholesterol ester, cholesterol, triacylglycerol, and lactosylceramide, known to influence both cholesterol sequestration and cell death. The study also shows that not only does the infection provide a strong stimulus, but that the tissue environment shapes macrophage gene expression significantly.

Genomic studies of host responses to tuberculosis by Thuong, Hawn *et al*. 161, show that macrophages from individuals with different clinical manifestations of *Mycobacterium tuberculosis* have distinct gene expression profiles and polymorphisms linked to susceptibility. The authors identified CCL1 as biomarker to distinguish the clinical groups and carriage of 6 single nucleotide polymorphisms correlated with infection in a case–control genetic association study with different clinical outcomes 161. Other transcriptome studies dedicated to microglia, placental and Hofbauer macrophages, adipose tissue and Kupffer cells are summarized in *Table *2.

**Table 2:** Selection of whole genome studies in tissue macrophages

Tissue/organ	Study title	Species	Geo Dataset identifier
Monocytes	Transcriptional profiling of CD16^+^ and CD16^−^ peripheral blood monocytes from healthy individuals Transcription and enhancer profiling in human monocyte subsets Transcriptome profiles of mouse and human monocyte and dendritic cell subsets	Human	GSE16836 GSE40502 GSE35459
	Comparison of gene expression profiles between human and mouse monocyte subsets Transcriptome profiles of mouse and human monocyte and dendritic cell subsets Murine blood monocyte subsets Microarray analysis of splenic reservoir monocytes and their blood counterparts	Mouse	GSE17256 GSE35459 GSE32364 GSE14850
Brain	Genome-Wide Expression Profiling of human tumor infiltrating microglia macrophages. Identification of a Unique TGF-β Dependent Molecular and Functional Signature in Microglia	Human	GSE25289 GSE48579
	Integrated expression profiles of mRNA and miRNA in polarized primary murine microglia	Mouse	GSE49331
Lung	Cigarette smoking effect on alveolar macrophage Alveolar macrophage response to bacterial endotoxin lipopolysaccharide exposure *in vivo* Alveolar macrophages of cigarette smokers	Human	GSE2125 GSE40885 GSE8823
	Genome-wide transcriptional analysis of tissue macrophages and bone marrow-derived macrophages (BMDMs)	Mouse	GSE56682
CVS	Similarities and differences in the transcriptome of human atherosclerotic and non-atherosclerotic macrophages Atherosclerotic Coronary Artery Disease: circulating mononuclear cell types	Human	GSE7074 GSE9820
	Gene expression in cardiac macrophages Expression data from mouse model of plaque regression	Mouse	GSE53787 GSE24819
Adipose tissue	Genome-wide analysis of visceral adipose tissue CD14^+^ cells from obese and obese diabetic subjects	Human	GSE54350
	Genome-wide transcriptional analysis of tissue macrophages and bone marrow-derived macrophages (BMDMs) Expression data from mouse adipose tissue macrophage	Mouse	GSE56682 GSE53403
Liver	Similarities and differences in the transcriptome of human atherosclerotic and non-atherosclerotic macrophages	Human	GSE7074
	Genome-wide transcriptional analysis of tissue macrophages and bone marrow-derived macrophages (BMDMs)	Mouse	GSE56682
Gastrointestinal tract	Normal colonic *lamina propria* macrophage and dendritic cell populations	Mouse	GDS4369
Placenta	Placenta environment induces the differentiation of macrophages into multinucleated giant cells Regulatory role of multinucleated giant cells derived from placental CD14^+^ macrophages: Pathophysiological implications Gene expression profile of decidual macrophages and different in vitro M1 and M2 macrophage populations	Human	GSE29575 GSE38747 GSE30595

Macrophage gene expression at whole genome level has been carried out for different tissues and species. However, given the heterogeneity in platforms and isolation methods it is difficult to compare results. Nevertheless, here we wanted to point to interesting studies available in Geo Dataset, which give an idea of the diversity and responsiveness of tissue macrophages.

Although high throughput techniques have begun to shed light on different aspects of macrophage heterogeneity, further integration of gene expression in all its stages to function is indispensable. In particular, proteomics, lipidomics, and phenomic *in situ* analysis need to be combined with microarrays to clarify the role of genes, mutations, expression levels, splicing, expression and ultimately gene function in these cells.

## Conclusions

We have reviewed some of the properties that underlie resident and recruited monocyte/macrophage heterogeneity in different mouse organs and tissue environments. Although it is apparent that differential gene expression is crucial during macrophage differentiation and activation, we have hitherto not fully exploited the power of genome and proteome-wide analytic tools to catalog common as well as distinctive tissue macrophage genes, proteins, and functions in different environments. This is partly due to difficulties in macrophage extraction from solid tissues, and limited *in situ* single cell analysis of multiple candidate gene products. Furthermore, we have very little understanding of macrophage-specific-transcriptional regulators, except where particular tissue functions can be implicated, e.g. following the clearance of senescent erythrocytes and turnover of hemoglobin in splenic red pulp, bone marrow, and liver. The macrophage plasma membrane receptor profile within different tissues, which we have emphasized in this review, is likely to determine their distinct gene expression profile, responding to information from other cells and ligands in each particular environment. Above all, we are ignorant of stromal-macrophage interactions, especially the nature and possible regional variation in stromal cells and extracellular matrix within different organs. This may benefit from novel culture models, and also from the experience of cancer cell biologists in studying tumor–host interactions *in vivo* and *in vitro* 162.

Improved understanding of phenotypic heterogeneity will guide our attempts to manipulate and target macrophage subpopulations selectively, an unmet need in disease prevention and treatment. The increasing promise of monoclonal antibody therapy depends on systematic study in mouse models and extensive validation in humans. This will require better monoclonal antibodies for immunocytochemistry of human macrophages in normal tissue and disease and improved methods to analyze their complexity.
